# Precision Food Composition Data as a Tool to Decipher the Riddle of Ultra-Processed Foods and Nutritional Quality

**DOI:** 10.3390/foods13081259

**Published:** 2024-04-19

**Authors:** Antonis Vlassopoulos, Alexandra Katidi, Stamoulis Noutsos, Maria Kapsokefalou

**Affiliations:** Laboratory of Chemistry and Food Analysis, Department of Food Science and Human Nutrition, Agricultural University of Athens, 11855 Athens, Greece; alexandra.katidi@gmail.com (A.K.); stam.nouts@gmail.com (S.N.); kapsok@aua.gr (M.K.)

**Keywords:** ultra-processed foods, NOVA, formulation, composition, ingredients, food composition database

## Abstract

Background: Epidemiology supports a link between ultra-processed foods (UPFs) and health, mediated mainly through the clustering of foods with suboptimal nutrient profiles within UPFs. However, successful NOVA categorization requires access to a food’s ingredient list, which we hypothesized can impact both UPF identification and the link between processing and composition. Methods: Foods (n = 4851) in the HelTH branded food composition database were classified as NOVA1-4, with or without using the ingredient lists (generic and branded approach, respectively), to identify differences in NOVA classification (chi-square test) and the estimated average nutritional composition of each NOVA group (Kruskal–Willis U test). Results: Using the ingredients list increased UPF identification by 30%. More than 30% of foods commonly assumed to be minimally processed (NOVA1-plain dairy, frozen vegetables, etc.) were reclassified as UPFs when using ingredient lists. These reclassified foods, however, had nutritional compositions comparable to NOVA1 foods and better than UPFs for energy, fat, sugars, and sodium (*p* < 0.001). In fact, UPFs did not show a uniform nutritional composition covering foods from Nutri-Score A (~10%) to Nutri-Score E (~20%). Conclusions: The assumption that all UPFs have the same unfavorable nutritional composition is challenged when NOVA is applied using the appropriate branded food composition database.

## 1. Introduction

Since the industrial revolution, packaged and processed foods have played an increasingly larger role in our daily diets, with today’s diets containing 60–70% of daily energy from processed and ultra-processed foods [1,2]. Although the detrimental effect of high fat, high sugar, and high salt diets on health have been known for decades [3,4,5], currently scientists are asked to understand whether processing per se may have an impact on health [6,7,8,9,10,11]. 

The NOVA system, the most commonly used system to study the impact of processing on health, assumes that all industrially produced foods are formulated with the sole aim of being highly palatable and hence that products of the same subcategory have the same formulation and therefore the same nutritional composition, e.g., all pizzas and all biscuits are equally rich in fat, sugar, salt, and energy [12]. Based on this rationale, the NOVA system classifies foods into four categories: minimally (un)processed, culinary ingredient, processed, and ultra-processed foods (UPFs), with the latter being any food that has been industrially manufactured, includes additives, and has a long shelf life. Although a number of other systems have been proposed to classify the degree of processing, the NOVA system has attracted significant interest, as a number of epidemiological studies have highlighted a detrimental relationship between NOVA4 food consumption and health, such as larger weight gains and increased risk of cardiovascular disease, cancer, etc. [13,14,15]. 

However, scientists are questioning whether the NOVA system has in fact the discriminatory capacity required to identify an effect of food processing independent of the well-established link between a food’s nutritional composition and health [16,17]. If in fact a link between processing and health exists, it is important to understand whether the NOVA system is a suitable tool to decipher this or whether it mediates its effects through the nutritional composition of foods. Previous reports from our group showed that, indeed, there was great agreement between the NOVA system and the Mediterranean Diet Pyramid, meaning that NOVA4 foods were more likely to be found in the top of the pyramid [18]. However, both our reports and studies in the UK showed that the link between NOVA groups and nutritional composition is not always straightforward, as not all NOVA4 foods/UPFs have suboptimal nutritional composition [18,19]. 

We hypothesized that the main source of disagreement in the literature is in fact linked with the food composition datasets used in the published literature. Specifically, cohort and other epidemiological studies measure food exposure using FFQs and 24 h recalls, which are analyzed using generic food composition databases, i.e., databases that provide information only on the nutritional composition and the name of a food, and the presence of additives, the degree of processing, and other key NOVA input variables have to be assumed instead of being provided. The creators of the NOVA system have themselves identified issues in applying it on generic databases that do not offer information on how these foods were prepared and their specific composition [13]; these problems are experienced even by experts in the field of nutrition [20]. On the other hand, the appropriate application of NOVA on a food composition database would require access to the full ingredient list of a food to successfully assign a classification without speculations. Branded food composition databases offer this type of information, and in our previous analyses, we showed that even foods like plain yogurt, commonly considered a NOVA1 (minimally processed food), could actually be classified as NOVA1 or NOVA4 if researchers had access to the full ingredient list [18]. 

In the current study, we aimed to directly investigate the impact of having access to branded food data when applying the NOVA system on food classification itself and the related assumptions about the nutritional value of foods with different degrees of processing. To achieve this, we used the HelTH branded food composition database available in Greece, and we developed two NOVA classification approaches: one mimicking the system’s application on generic food composition databases, using only the product’s name as an input, and another employing the product’s name and ingredient list available in a branded food composition database. The two approaches, generic versus branded, were compared to identify i) differences in NOVA classification between the two and ii) whether these differences in NOVA classification were linked to differences in the nutritional quality of foods assigned to each NOVA group. 

## 2. Materials and Methods

### 2.1. Data Source

The Hellenic Food Thesaurus (HelTH), in the Greek Branded Food Composition Database (BFCD), was the data source for the current study. The latest version of HelTH (n = 4851), after a targeted expansion towards plant-based protein sources, was used. Briefly, HelTH is a database that includes food data as presented on-package, collected through the online sampling of the foods available in the main online supermarkets in Greece and curated by trained compilers. In the current study, the food data used included the nutritional declaration, the ingredient list, and all data presented on the package, which allowed the product identification, categorization in food groups and subgroups, and specification of the food’s physical state and/or the manufacturing processes used in its production. 

### 2.2. Classification of Foods Using the NOVA System Groups

All foods in the HelTH database were classified into four distinct groups, according to the level and purpose of processing as in the NOVA system. (i) NOVA1—unprocessed or minimally processed foods: This includes all foods that are directly taken from nature without any processing or with minimal processing or preservation. It includes both animal- and plant-based foods that have no added ingredients. (ii) NOVA2—culinary ingredients: This includes the salt, sugar, oils, and starch that are derived from unprocessed foods or minimally processed foods (e.g., olive oil, flour). (iii) NOVA3—processed foods: This includes all foods produced through traditional processing techniques, which add culinary ingredients to an unprocessed/minimally processed food (e.g., freshly baked breads, canned vegetables, or cured meats). (iv) NOVA4—UPFs: This includes all ready-to-eat industrially formulated products that include additives and/or substances extracted from foods but contain little to no intact unprocessed/minimally processed ingredients. 

For the current study, two approaches for NOVA classification were developed. 

Approach 1: NOVA classification based on the product name (generic food composition approach)

The first approach aimed to mimic the application of NOVA on generic food composition databases. For this classification, only the product’s name and basic descriptors were extracted from the front of the packaging. These details are recorded as Product Name and Long Name in the HelTH database. Namely, the variable Product Name includes the name of the product exactly as mentioned at the supermarket’s online platform, without any specific curation. The Long Name variable includes a short description of the product, containing the manufacturer’s name, the product name, the food group, basic characteristics of the product, and the package size. The terms used to decide on the NOVA classification are presented in detail in Table 1.

Approach 2: NOVA classification based on product name and ingredient list (branded food composition approach)

HelTH allows access to all the information needed to identify any additives or ingredients that would justify a reclassification of a food most likely under the group NOVA4, and so ingredient lists were searched for the presence of caloric and/or noncaloric sweeteners in their many forms, added sodium in their many forms, and added oils. Additional searches were conducted for ≥1 mention of protein isolates or concentrates; added natural flavors and flavor enhancers; emulsifiers; bulking agents and other thickeners, such as sodium carboxymethyl cellulose, cellulose gel, guar gum, xanthan gum, carrageenan, etc.; and a variety of antioxidants, preservatives, and ingredients rarely used in kitchens, such as vitamin A palmitate, vitamin D2, zinc sulfate, sulfur dioxide, etc. Products whose ingredient lists could not be acquired were excluded (n = 27). All the remaining products of the HelTH BFCD (n = 4824) were classified into one of the four NOVA groups based on their ingredient list.

**Table 1:** List of indicative terms found in the product name of generic foods and their respective use to classify foods into NOVA groups.

NOVA1	-natural, packaged, cut, chilled, or frozen -bulk or packaged grains -fresh or pasteurized vegetables or fruit juices with no added sugar or other substances -grains or wheat, oats, and other cereals -grits, flakes, and flours made from corn, wheat, or oats, including those fortified with iron, folic acid, and other nutrients lost during processing -dried or fresh pasta, couscous, and polenta made from water and the grits/flakes/flours described above -eggs -lentils, chickpeas, beans, and other legumes -dried fruits -nuts, peanuts, and other seeds without salt or sugar -fresh or dried herbs and spices (e.g., oregano, pepper, thyme, cinnamon) -fresh and dried mushrooms and other fungi or algae -fresh, chilled, or frozen meat, poultry, fish, and seafood, whole or in the form of steaks, fillets, and other cuts -fresh or pasteurized milk; yoghurt without sugar -tea, herbal infusions, coffee -tap, spring, and mineral water
NOVA2	-oils made from seeds, nuts, and fruits, including soybeans, corn, oil palm, sunflower, or olives -white, brown, and other types of sugar and molasses obtained from cane or beet -honey extracted from honeycombs -syrup extracted from maple trees -starches extracted from corn and other plants -butter -lard -coconut fat -refined or coarse salt, mined or from seawater -any food combining 2 of these, such as “salted butter”
NOVA3	-canned or bottled legumes or vegetables preserved in salt (brine) or vinegar, or by pickling -tomato extract, pastes, or concentrates (with salt and/or sugar) -fruits in sugar syrup (with or without added antioxidants) -beef jerky, bacon -salted or sugared nuts and seeds -canned fish, such as sardines and tuna, with or without added preservatives -salted, dried, smoked, or cured meat or fish -coconut fat -freshly made (unpackaged) breads made of wheat flour, yeast, water, and salt -fermented alcoholic beverages such as beer, alcoholic cider, and wine
NOVA4	-fatty, sweet, savory, or salty packaged snacks -biscuits (cookies), chocolates, candies, and confectionery in general -ice creams and frozen desserts -cola, soda, and other carbonated soft drinks -”energy” and sports drinks -canned, packaged, dehydrated (powdered), and other “instant” soups, noodles, sauces, desserts, drink, mixes, and seasonings -sweetened and flavored yogurts, including fruit yogurts -dairy drinks, including chocolate milk -sweetened juices -margarines and spreads -pre-prepared (packaged) meat, fish, and vegetables, pizza, pasta dishes, burgers, hot dogs, sausages, poultry, and fish “nuggets” and “sticks” -other animal products made from remnants -packaged breads, hamburger and hot dog buns -baked products made with ingredients such us hydrogenated vegetable fat, sugar, yeast, whey, emulsifiers, and other additives -breakfast cereals and bars -infant formulas and drinks, meal replacement shakes (e.g., “Slim Fast”) -pastries, cakes, and cake mixes -Industrial formulations and manufacturing techniques such as extrusion, molding, and preprocessing by frying

### 2.3. Application of the Nutri-Score Algorithm

The Nutri-Score algorithm was calculated for each food based on its nutritional composition per 100 g/mL of food/beverage, as previously described [18,21,22,23]. Briefly, Nutri-Score studies energy (kJ), total sugars (g), saturated fatty acids (SFAs) (g), and sodium (mg) as “negative nutrients” and scores them on a scale from 0 to 10 for increasing content. On the other hand, protein (g), fiber (g), and fruits/vegetables/pulses/nuts/oils (FV%), the “positive nutrients”, are scored from 0 to 5 for increasing content. “Negative” and “positive” nutrient scores are combined to calculate the FSAm-NPS score (Range: −15 to +40) by subtracting the “positive nutrients” score from the “negative nutrients” score. Apart from the numerical FSAm-NPS score, each food is given a Nutri-Score grade from A to E (five-point Nutri-Score) based on the following criteria: (A) is given to solid foods with FSAm-NPS scores from −5 to −1 and only to waters among beverages, (B) is given to solid foods with FSAm-NPS scores from 0 to 2 and beverages with FSAm-NPS scores from −15 to 1, (C) is given to solid foods with FSAm-NPS scores from 3 to 10 and beverages with FSAm-NPS scores from 2 to 5, (D) is given to solid foods with FSAm-NPS scores from 11 to 18 and beverages with FSAm-NPS scores from 6 to 9 and (E) is given to solid foods with FSAm-NPS scores from 19 to 40 and beverages with FSAm-NPS scores from 10 to 40.

Missing data for any of the “negative nutrients” (energy, saturated fat, total sugar, or sodium) led to an inability to calculate an FSAm-NPS score, and the respective Nutri-Score Grade and such foods were excluded from the analysis (n = 877). On the contrary, missing data for any “positive nutrients” was imputed with zero, and the FSAm-NPS score and Nutri-Score grade calculations were performed accordingly. Data imputation for “positive nutrients” took place for <10% of foods in food groups where such nutrients are relevant. The main sources of missing nutrient values were lack of nutritional declaration or inability to obtain data due to the low quality of the available images.

### 2.4. Statistical Analysis

Statistical analysis was carried out using IBM SPSS Statistics^®^ (version 23, Northridge, CA, USA). Nutritional composition data (content per 100 g or 100 mL of product) and the FSAm-NPS score were analyzed as continuous variables. Data were tested for normality using the Kolmogorov–Smirnov test. None of the variables followed the normal distribution. Therefore, variables were expressed as median (interquartile range). Differences in the NOVA group and Nutri-Score grade distribution were tested using the chi-square test. Differences in the nutritional composition and the FSAm-NPS score were tested using the Kruskal–Wallis non-parametric test for k independent samples. Between-group differences were tested using the Mann–Whitney U test for continuous variables. Statistical significance was set at 5%, and Bonferroni correction was applied for multiple comparisons. 

## 3. Results

### 3.1. Differences in NOVA Classification between the Generic and Branded Food Approaches

Using either method for NOVA classification >50% of the packaged foods available in the HelTH database were classified as NOVA4. However, having access to the ingredient list significantly impacted the NOVA group distribution, resulting in a 1.3-fold increase in the NOVA4 foods. In fact, when the ingredients list was checked, a number of foods that were classified via the name only as NOVA 1, 2, or 3 were now classified as NOVA4, namely 32.5% of foods classified as NOVA1, 11.5% of foods classified as NOVA2, and 62.2% of foods classified as NOVA3 via the name only were reclassified as NOVA4. The only other reclassification observed was that of NOVA1 foods to NOVA3, which was the case for 97 foods (9.1% of NOVA1 foods based on the generic approach). 

Only the NOVA classification of eggs and ready meals was not impacted by having access to the ingredient list. The largest impact was seen for meat and meat products, with almost all products being classified as NOVA4 using the ingredient list (2.2-fold increase) (Table 2), with most of the changes taking place among preserved meats and meat dishes (Table 3). Similarly, fruits were greatly impacted; using a generic food approach, all fruits were classified as NOVA1 or NOVA3, but having access to the ingredient list meant that 86.5% of fruits were grouped as NOVA4 (Table 2). Screening the ingredient list was linked to 90.9%, 90%, 57.1%, 35.5%, and 43.8% of NOVA1 foods being reclassified as NOVA4 in Miscellaneous foods, Fruits and Fruit Products, Beverages, Dairy Products, and Nuts and Seeds, respectively. 

As far as food subcategories are concerned, having access to the ingredient list reclassifies almost the totality (>90%) of fruit products (including dried fruit), preserved meats, and sausages to NOVA4 from either NOVA1 or NOVA3 (Table 3). Similarly, in order to correctly identify NOVA4 nuts, the ingredient list was necessary for 87.5% of them. Without access to the ingredient list, 34.4% of NOVA4 nuts would be classified as NOVA1. The same was true for juices, where access to the ingredient list doubled the number of juices classified as NOVA4. Interestingly, even the classification of milk and yogurt products requires access to the ingredient list, as ~35% of NOVA4 milks and yogurts would be misclassified as NOVA1 without the ingredient list. For cheese, the importance of having access to the ingredient list is even greater, as the proportion of cheeses classified as NOVA4 instead of NOVA3 after inspecting the ingredient list is four times larger.

### 3.2. Differences in the Nutritional Quality of NOVA Groups as Identified via the Generic versus the Branded Food Approach

#### 3.2.1. Differences in the Nutritional Composition

**Figure 1:**
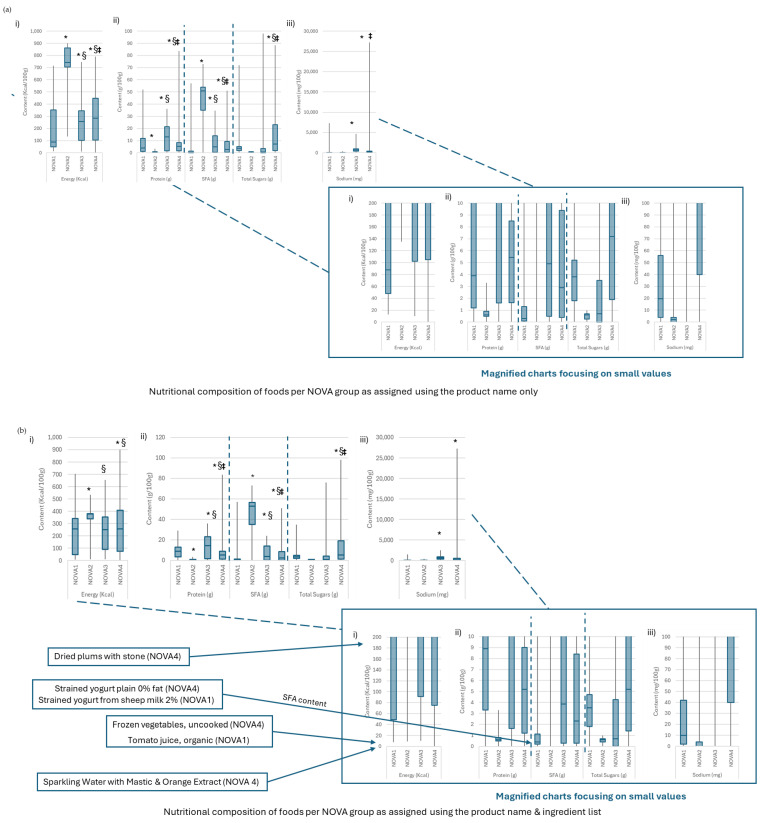
(**a**,**b**) Nutritional composition per 100 g for energy (**i**), protein, saturated fats (SFA), total sugars (**ii**) and sodium (**iii**) per NOVA group as assigned (**a**) using only the product name and (**b**) using both the product name and ingredient list. Differences in distribution were tested using the Kruskal–Wallis test. Pairwise comparisons were carried out using the Mann–Whitney U with Bonferroni correction. * Indicates *p*-value < 0.001 vs. NOVA1, § indicates *p*-value < 0.001 vs. NOVA2, ‡ indicates *p*-value < 0.001 vs. NOVA3. Magnified view of the low content values (near-zero values) for clarity provided at the bottom of each graph. Indicative examples presented for foods that were reclassified using the branded approach and their non-reclassified counterparts (for illustration purposes).

**Figure 2:**
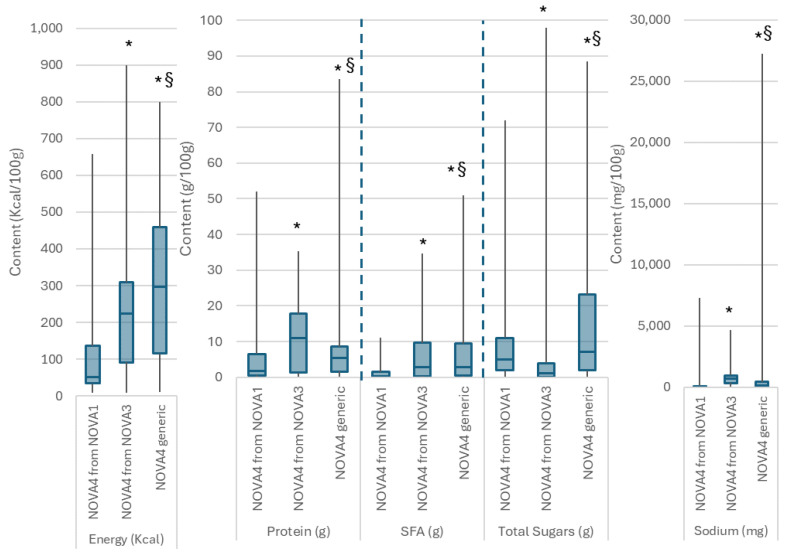
Nutritional composition per 100 g for energy, protein, saturated fats (SFA), total sugars and sodium for foods identified as NOVA4 based on the product name (generic) and products reclassified as NOVA4 after searching the ingredient list (originally grouped as NOVA1 or NOVA3 based on their name). Pairwise comparisons carried out using the Man-Whitney U with Bonferroni correction. * Indicates *p*-value < 0.02 vs. NOVA4 from NOVA1, § indicates *p*-value < 0.001 vs. NOVA4 from NOVA3.

As shown in Figure 1, irrespective of the method used to assign foods in NOVA groups, on average, NOVA1 foods had lower energy, total sugars, and saturated fat content and higher protein content. However, overall, NOVA2 foods were those with the highest saturated fat and energy content among all, as NOVA3 foods had the highest protein content. Moving from a generic approach to NOVA classification to a branded approach made the differences between the energy and sodium content of NOVA3 foods compared to NOVA4 foods less apparent. As shown in Figure 2, foods that were reclassified to NOVA4 from the NOVA1 and NOVA3 categories after an ingredient list search had better nutritional composition in terms of energy, saturated fats, total sugars, and sodium compared to the NOVA4 foods identified through the name only. As far as protein is concerned, the highest protein content was observed among foods that were classified as NOVA3 based on their name and were reclassified to NOVA4 based on their ingredients. Similarly, the lowest protein content was found in former NOVA1 foods reclassified as NOVA4. 

This was more pronounced among specific food categories, such as dairy products and grain products, for which the use of the ingredient list was associated with the reclassification of multiple NOVA1 foods as NOVA4 despite their better nutritional composition. On the other hand, in the same categories, NOVA3 foods that were reclassified as NOVA4 tended to have higher content of almost all nutrients (Table 4).

When the NOVA system was applied using only the product name, a positive relationship between the FSAm-NPS Score and the NOVA groups was seen (*p* < 0.001), meaning that with increased processing the FSAm-NPS Score decreased. More specifically, NOVA1 foods (minimally processed) had a significantly better FSAm-NPS Score than all other NOVA groups (*p* < 0.001), but no other pairwise differences were seen. The same was true when NOVA classification was obtained using both the product name and the ingredient list, with the difference that having access to the ingredient list was linked to NOVA4 foods exhibiting a higher FSAm-NPS Score compared to NOVA3 foods (*p* = 0.004).

#### 3.2.2. Differences in Nutri-Score Performance

**Figure 3:**
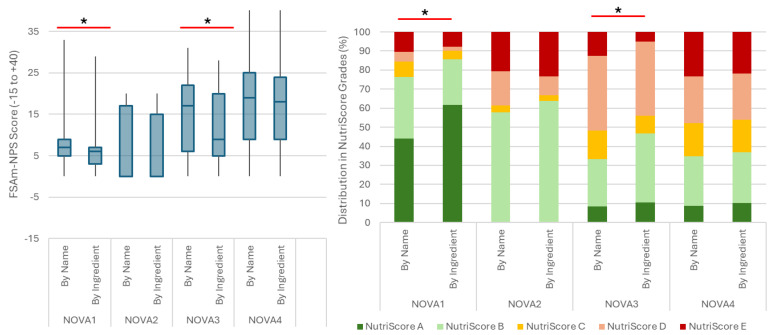
Performance in the Nutri-Score system expressed as FSAm-NPS score and Nutri-Score grade per NOVA group and per NOVA classification methodology, using either only the product name (by name) or a combination of the product name and the ingredient list (by ingredient) as input. * Indicates *p*-value < 0.001 for pairwise comparisons by name and by ingredient within the same NOVA group either using the Kruskal–Wallis test (**left**) or the chi-square test (**right**).

This could be explained by the shifts in FSAm-NPS score introduced by the reclassification of foods based on the ingredient list. Food reclassification was linked to an improved FSAm-NPS score for NOVA1 and NOVA3 foods (Figure 3, left). This was also seen as Nutri-Score grade distribution towards the lower grades for all NOVA groups following food reclassification (Figure 3, right). In fact, foods reclassified from NOVA1 to NOVA4 had a lower FSAm-NPS score than the foods already classified as NOVA4 from the product name and the foods reclassified from NOVA3 to NOVA4 (*p* < 0.001 for both). No difference in the FSAm-NPS score was seen between the foods reclassified from NOVA3 to NOVA4 and those classified as NOVA4 already from the product name (*p* = 0.99). 

Most foods reclassified as NOVA4 from NOVA1 (61.1%) after assessing the ingredient list were assigned Nutri-Score A or B. The same was observed only in 31% of NOVA4 foods reassigned from NOVA3, indicating a closer resemblance between NOVA3 and NOVA4 foods in terms of their Nutri-Score performance (Table 5).

When foods were classified under NOVA based on the branded approach, NOVA1 had a significantly better Nutri-Score performance compared to NOVA4 foods. NOVA1 foods were assigned Nutri-Score grades mainly from A to B (with a few exceptions reaching up to C). On the contrary, NOVA4 foods were assigned Nutri-Score grades from A to E, with the distribution in each grade differing between food subcategories (Table 6). For example, in dairy products and grain products (except fine bakery products and savory cereals), the majority of NOVA4 foods were assigned Nutri-Score grades from A to C, similar to NOVA1 foods (Table 6). 

## 4. Discussion

To our best knowledge, this study is the first to directly investigate the impact of having access to branded food data when applying the NOVA classification system.

In our analysis, it becomes apparent that without access to the ingredients list, up to 20.5% of foods in total are misclassified in NOVA groups. When foods were treated as generic foods, only 54% of them were classified as NOVA4, a number that increased to 70% when foods were treated based on their ingredient list. This effect is more apparent in certain food categories than others. The only food groups not affected by having access to the ingredient list are breakfast cereals, preserved meats, meat imitations, seafood products, ready meals, chocolate confectionery, jams, and honey. On the contrary, 42.8% of foods that the generic approach would classify as NOVA1 (minimally processed) and would hence be linked to health benefits would in fact be classified as NOVA4 (UPFs) if the researchers had access to the ingredient list. These include unflavored milk and yogurt, dried fruit and nuts, vegetable products, and even rice and pasta. The extend of misclassification is even more apparent in the NOVA3 groups, as 63.8% of foods classified as NOVA3 with the generic approach would be reclassified to NOVA4 using a branded approach.

As far as nutritional composition is concerned, the most interesting finding is that NOVA4 foods have a great variability in their macronutrient content, which goes against the NOVA system’s assumptions that all UPFs have a high sugar, fat, and sodium content. In fact, NOVA4 foods do have a higher content of these nutrients compared to their NOVA1 counterparts, but this is not true when compared to NOVA3 foods. The application of the NOVA system using the ingredients list, as required by the system’s definition, increases the variability of the UPFs’ nutritional composition, as many foods are reclassified from NOVA1 and NOVA3 categories. These reclassifications result in extremely limited differences in the nutritional composition of NOVA3 and NOVA4 foods. This is an important finding, as the NOVA system and its associated literature indicate health risks solely for UPFs (NOVA4) [13,14,15], when in fact homemade foods, artisanal products, and all out-of-home, delivery, and take-out foods (NOVA3) are likely to have the same nutritional composition as their industrially produced counterparts. It is yet unclear whether a potential shift from NOVA4 to NOVA3 is likely to result in better nutritional quality of the overall diet based on our results. What is more, penalizing all UPFs equally based on data poorly equipped to describe the foodscape can lead to reduced consumption of food groups such as dairy, cereals, fruits, and vegetables, which in the current market may be characterized as UPFs simply due to the addition of preservatives, without any impact on their nutritional composition. Apart from the public confusion linked to this, the impact of such policies could be exacerbated in sensitive populations such as people living with food insecurity or in food desserts, for which products with a long shelf life are a staple, and UPFs with high nutritional value may play an important role in their dietary intake [24,25]. 

Studying the overall nutrient profile of foods highlighted the same issues, with NOVA4 (UPFs) exhibiting a great variability of Nutri-Score grades, which was further expanded when the NOVA system was applied using a branded food composition database. This variability in Nutri-Score grading among UPFs is in agreement with previous research in the UK, Italy, and other European countries [17,19,26,27]. Nonetheless, it remains true that after the branded classification of the NOVA system, minimally processed foods, despite their small numbers, were all graded as either A or B. In the light of these results, it might be interesting to consider that although the NOVA system was designed to identify UPFs, it might actually be more successful in identifying minimally processed foods and, in fact, minimally processed foods with the optimal nutritional quality. This could offer a different read to the existing literature, showing a protective role of minimally processed foods on health, leaving room for a further investigation of the exact role of processed and ultra-processed foods on health. This further investigation needs to be able to ensure access to branded data to correctly apply the NOVA system and to dissect the effects between processing and formulation (nutritional composition). Recently, an analysis of the EPIC cohort showed that the link between UPFs and ill-health was only true for UPFs from the processed meats and sugar-sweetened beverage food categories [7]. The authors of that study alongside previous reports highlighted that although UPFs are believed to have a homogeneous nutritional composition [28], they are in fact extremely heterogeneous [29]. Other studies have also identified that UPFs from food categories such as grains could have positive associations with health, as they are often fortified with fiber [30]. 

Such datasets will not only be useful in epidemiology but could help the design of clinical trials. To date, only one clinical trial has been designed to study the effect of a UPF-rich diet on health [31], but the intervention and control diets failed to mimic each other in terms of nutritional composition. Hence, the results cannot be linked to processing alone. With the use of a branded food composition database, researchers can identify foods within the same (sub)category with similar nutritional composition but with different NOVA classification, and as such they can successfully design almost identical diets for the control and intervention arms. 

Overall, future research should aim to understand if the NOVA system is another expression of existing systems to classify foods based on their nutritional value, such as the Mediterranean Diet Pyramid or terms like “junk” food and Western Diet, or if it indeed adds a new dimension to the concept of nutritional value, that of the degree of processing. To achieve this, more mechanistic data will be required, linking additives and specific processes to ill-health beyond their impact on the nutritional composition. It will also be important to identify whether all UPFs, with their varying nutritional composition, have the same impact on health, and if not, the NOVA system should be adapted to address these issues, potentially by adopting a separate set of criteria for nutritional composition [32]. 

Although applying the NOVA system on a branded food database is the main strength and novelty of this study, using such a dataset also introduces certain limitations. HelTH, like all branded food composition databases, includes data on specific nutrients required to be present in the nutritional declaration, meaning that analyses of the micronutrient and fiber content were not possible. Also, HelTH, despite its extent does not include all foods sold in Greek supermarkets. Products from smaller producers as well as artisanal and local foods are likely to be misrepresented, and data on raw, unpackaged foods are completely missing. However, the addition of these foods are unlikely to impact the results, as raw foods would be classified as NOVA1 by definition. It would be interesting, however, to map the distribution of local and artisanal produce among the NOVA3 and NOVA4 categories and understand the manufacturing practices employed from smaller enterprises and their link to nutritional value. 

## 5. Conclusions

The current study highlights that not having access to branded food composition data is likely to lead to NOVA misclassification. Although the NOVA system is devised to split foods into minimally processed, processed, and ultra-processed, which would be linked to significant differences in their nutritional composition relevant to public health, when applied using granular food data, these differences were not seen. The assumption that all UPFs have the same unfavorable nutritional composition (high in fat, sugar, and salt) is challenged when NOVA is applied using the appropriate branded food composition database, as UPFs show wide heterogeneity in their nutritional composition. This heterogeneity is likely to remain unseen in epidemiological surveys that do not use branded food data. The utilization of branded food composition databases in nutrition research could be pivotal in disentangling the mixed effects of degree of processing and nutritional quality on health and to better guide public health interventions. 

## Figures and Tables

**Table 2:** Distribution of foods in NOVA groups using either only the product name (by name) or a combination of the product name and the ingredient list (by ingredient) as input, per food category.

FOOD CATEGORY	NOVA CLASSIFICATION								
	*By Name*				*By Ingredients*				*p*-Value
	NOVA1	NOVA2	NOVA3	NOVA4	NOVA1	NOVA2	NOVA3	NOVA4	
**Dairy and Substitutes**	365 (34)	0 (0)	264 (25)	432 (41)	146 (14)	0 (0)	187 (18)	715 (68)	**<0.001**
**Egg and Egg Products**	35 (97)	0 (0)	0 (0)	1 (3)	34 (94)	0 (0)	0 (0)	2 (6)	Ν/A
**Meat and Meat Products**	8 (3)	0 (0)	129 (52)	109 (44)	1 (<1)	0 (0)	2 (1)	237 (99)	**<0.001**
**Seafood, Fish and Products**	0 (0)	0 (0)	73 (89)	9 (11)	0 (0)	0 (0)	54 (67)	27 (33)	**<0.001**
**Fats and Oils**	0 (0)	42 (52)	0 (0)	39 (48)	0 (0)	33 (41)	0 (0)	48 (59)	**<0.001**
**Grains and Grain Products**	276 (24)	0 (0)	17 (1)	849 (74)	235 (26)	0 (0)	12 (1)	894 (78)	**<0.001**
**Nuts, Seeds, and Kernels**	62 (47)	0 (0)	58 (44)	11 (8)	29 (22)	0 (0)	34 (26)	67 (52)	**<0.001**
**Vegetables and Vegetable Products**	539 (88)	0 (0)	50 (8)	27 (4)	489 (80)	0 (0)	37 (6)	88 (14)	**<0.001**
**Fruit and Fruit Products**	20 (44)	0 (0)	25 (56)	0 (0)	1 (2)	0 (0)	5 (11)	38 (86)	0.335
**Sugars and Sugar Products**	2 (<1)	35 (9)	47 (12)	321 (79)	1 (<1)	35 (9)	28 (7)	341 (84)	**<0.001**
**Beverages**	119 (27)	0 (0)	0 (0)	329 (73)	48 (11)	0 (0)	1 (<1)	397 (89)	**<0.001**
**Ready Meals**	0 (0)	0 (0)	0 (0)	84 (100)	0 (0)	0 (0)	0 (0)	84 (100)	Ν/A
**Miscellaneous**	5 (1)	1 (<1)	57 (12)	406 (87)	5 (1)	1 (<1)	7 (1)	460 (97)	**<0.001**
**TOTAL**	**1431 (29.5)**	**78 (1.6)**	**720 (14.9)**	**2617 (54.0)**	**989 (20.4)**	**69 (1.4)**	**367 (7.6)**	**3398 (70.5)**	**<0.001**

Data presented as n (%), *p*-value for changes in the distribution of foods in NOVA groups using the by-name vs. by-ingredients approach using the chi-squared test.

**Table 3:** Distribution of foods in NOVA groups using either only the product name (by name) or a combination of the product name and the ingredient list (by ingredient) as input, per food subcategory.

FOOD SUBCATEGORY	NOVA CLASSIFICATION									
	*By Name*				*By Ingredients*				*p*-Value	
	NOVA1	NOVA2	NOVA3	NOVA4	NOVA1	NOVA2	NOVA3	NOVA4		
**Cream**	0	0	0	50 (100%)	0	0	0	50 (100%)	N/A	
**Milk**	122 (69.7%)	0	6 (3.4%)	47 (26.9%)	94 (54%)	0	6 (3%)	74 (43%)	**<0.001**	
**Yoghurt**	88 (51.2%)	0	0	84 (48.8%)	46 (27.2%)	0	0	123 (72.8%)	**<0.001**	
**Cheese**	0	0	191 (87.6%)	27 (12.4%)	0	0	127 (59.6%)	86 (40.4%)	**<0.001**	
**Imitation Milk Products**	155 (37.4%)	0	67 (16.2%)	192 (46.4%)	6 (1.4%)	0	54 (13.2%)	350 (85.3%)	**<0.001**	
**Frozen Dairy Desserts**	0	0	0	40 (100%)	0	0	0	40 (100%)	N/A	
**Fresh or Processed Eggs**	35 (100%)	0	0	0	34 (100%)	0	0	0	N/A	
**Poultry Meat**	3 (37.5%)	0	0	5 (62.5%)	0	0	0	8 (100%)	N/A	
**Meat Analogue**	0	0	0	111 (100%)	0	0	0	111 (100%)	N/A	
**Preserved Meat**	1 (1.2%)	0	82 (98.8%)	0	1 (1.2%)	0	2 (2.4%)	80 (96.4%)	**<0.001**	
**Sausage or Similar Meat**	0	0	38 (100%)	0	0	0	0	37 (100%)	N/A	
**Meat Dish**	5 (20%)	0	8 (32%)	12 (48%)	0	0	0	20 (100%)	0.056	
**Seafood or Related Organism**	0	0	5 (100%)	0	0	0	2 (40%)	3(60%)	**<0.001**	
**Seafood Product**	0	0	73 (91.3%)	7 (8.7%)	0	0	54 (68.4%)	25 (31.6%)	N/A	
**Vegetable Fat or Oil**	0	8 (100%)	0	0	0	1 (12.5%)	0	7 (87.5%)	N/A	
**Margarine or Lipid of Mixed Origins**	0	0	0	39 (100%)	0	0	0	39 (100%)	N/A	
**Butter or Other Animal Fat**	0	34 (100%)	0	0	0	32 (94%)	0	2 (6%)	N/A	
**Cereal or Cereal-Like Milling Products and Derivatives**	0	0		51 (100%)	0	0	0	51 (100%)	N/A	
**Rice or Other Grain**	76 (78.5%)	0	2 (2%)	19 (19.5%)	64 (66%)	0	2 (2%)	31 (32%)	**<0.001**	
**Pasta and Similar Products**	185 (91.1%)	0	14 (6.9%)	4 (2%)	167 (82.7%)	0	9 (4.5%)	26 (12.8%)	**<0.001**	
**Breakfast Cereals**	15 (9.5%)	0	1 (0.5%)	139 (90%)	4 (2.5%)	0	1 (0.5%)	150 (97%)	**<0.001**	
**Bread and Similar Products**	0	0	0	259 (100%)	0	0	0	259 (100%)	N/A	
**Fine Bakery Ware**	0	0	0	289 (100%)	0	0	0	289 (100%)	N/A	
**Savoury Cereal Dish**	0	0	0	88 (100%)	0	0	0	88 (100%)	N/A	
**Nuts**	36 (52.2%)	0	29 (42%)	4 (5.8%)	20 (29.5%)	0	16 (23.5%)	32 (47%)	**<0.001**	
**Seeds and Kernels**	11 (37.5%)	0	24 (68.5%)	0	0	0	18 (51.5%)	17 (48.5%)	0.803	
**Nut or Seed Product**	15 (55.5%)	0	5 (18.5%)	7 (26%)	9 (33.5%)	0	0	18 (66.5%)	**<0.001**	
**Vegetable (Excluding Potato)**	112 (65.1%)	0	50 (29.1%)	10 (5.8%)	65 (38.2%)	0	35 (20.6%)	70 (41.2%)	**<0.001**	
**Starchy Root or Potato**	4 (19%)	0	0	17 (81%)	1 (4.8%)	0	2 (9.5%)	18 (85.7%)	**<0.001**	
**Pulse and Pulse Product**	423 (100%)	0	0	0	423 (100%)	0	0	0	N/A	
**Processed Food Product (Fruit)**	20 (44.4%)	0	25 (55.6%)	0	1 (2%)	0	5 (11.5%)	38 (86.5%)	0.335	
**Sugar, Honey, or Syrup**	1 (2%)	35 (76%)	6 (13%)	4 (9%)	1 (2%)	35 (76%)	6 (13%)	4 (9%)	**<0.001**	
**Jam or Marmalade**	0	0	0	83 (100%)	0	0	0	83 (100%)	N/A	
**Non-Chocolate Confectionery or Other Sugar Product**	1 (1.5%)	0	41 (60.5%)	26 (38%)	0	0	22 (32.5%)	46 (67.5%)	**<0.001**	
**Chocolate or Chocolate Product**	0	0	0	208 (100%)	0	0	0	208 (100%)	N/A	
**Juice or Nectar**	114 (69%)	0	0	51 (31%)	48 (29.5%)	0	1 (0.5%)	114 (70%)	**<0.001**	
**Non-Alcoholic Beverage**	5 (2%)	0	0	278 (98%)	0	0	0	283 (100%)	N/A	
**Spice, Condiment, or Other Ingredient**	3 (1.1%)	1 (0.4%)	57 (20.1%)	223 (78.5%)	1 (0.4%)	1 (0.4%)	7 (2.5%)	275 (96.8%)	**<0.001**	
**Prepared Food Product**	2 (1.2%)	0	0	170 (98.8%)	0	0	0	172 (100%)	N/A	
**Ready-To-Eat Food**	0	0	0	43 (100%)	0	0	0	43 (100%)	N/A	
**Frozen, Semi-Ready Meal**	0	0	0	41 (100%)	0	0	0	41 (100%)	N/A	

Data presented as n (%), *p*-value for changes in the distribution of foods in NOVA groups using the by-name vs. by-ingredients approach using the chi-squared test.

**Table 4:** Nutritional composition per 100 g for energy, protein, saturated fats (SFA), total sugars, and sodium for foods identified as NOVA4 based on the product name (generic) and products reclassified as NOVA4 after searching the ingredient list (originally grouped as NOVA1 or NOVA3 based on their name).

	Energy (Kcal)			Protein (g)			SFA (G)			Total Sugars (g)			Sodium (mg)		
	NOVA4 from NOVA1	NOVA4 from NOVA3	NOVA4 Generic	NOVA4 from NOVA1	NOVA4 from NOVA3	NOVA4 Generic	NOVA4 from NOVA1	NOVA4 from NOVA3	NOVA4 Generic	NOVA4 from NOVA1	NOVA4 from NOVA3	NOVA4 Generic	NOVA4 from NOVA1	NOVA4 from NOVA3	NOVA4 Generic
**Dairy and Substitutes**	50.0 (32, 71)	279.0 (199, 337)	96.0 (63, 221)	1.4 (1, 5)	11.0 (1, 25)	3.2 (1, 5)	0.9 (0, 2)	18.0 (3, 21)	1.3 (1, 9)	3.7 (1, 5)	0.0 (0, 1)	7.1 (3, 12)	52.0 (40, 72)	700.0 (440, 800)	48.0 (40, 68)
**Meat and** **Meat Products**	196.0 (156, 238)	215.0 (125, 273)	222.0 (192, 248)	19.0 (15, 21)	14.6 (13, 22)	16.0 (14, 22)	4.7 (1, 6)	4.3 (1, 8)	1.8 (1, 5)	1.1 (1, 2)	1.0 (0, 1)	1.1 (1, 2)	-	952.0 (800, 1000)	600.0 (560, 680)
**Seafood, Fish, and Products**	-	187.0 (102, 341)	203.0 (195, 233)	-	11.6 (10, 15)	12.3 (12, 13)	-	2.1 (1, 6)	1.0 (1, 1)	-	1.0 (0, 1)	0.9 (1, 2)	-	628.0 (560, 804)	364.0 (360, 400)
**Grains and** **Grain Products**	358.0 (272, 402)	359.5 (290, 375)	399.0 (317, 467)	8.2 (7, 11)	12.6 (11, 13)	8 (6, 10)	0.7 (0, 2)	3.5 (2, 5)	4.9 (2, 9)	2.8 (1, 14)	3.7 (3, 5)	12.7 (3, 25)	216.0 (60, 340)	610.0 (520, 680)	320.0 (200, 500)
**Nuts, Seeds, and** **Kernels**	517.5 (402, 607)	574.0 (248, 625)	573.0 (568, 579)	14.9 (10, 21)	17.4 (2, 22)	18.4 (16, 24)	5.0 (4, 8)	6.0 (3, 8)	8.4 (8, 10)	5.3 (1, 11)	2.7 (0, 5)	7.4 (1, 31)	220.0 (16, 1400)	528.0 (208, 1700)	16.0 (0, 72)
**Vegetables and** **Vegetable Products**	20.0 (18, 29)	26.0 (25, 31)	136.0 (94, 195)	1.6 (1, 2)	1.5 (1, 2)	2.6 (2, 7)	0.1 (0, 0)	0.1 (0, 0)	0.6 (0, 1)	2.4 (0, 4)	3.5 (3, 4)	1.3 (1, 4)	28.0 (12, 252)	20.0 (12, 252)	52.0 (24, 208)
**Fruit and** **Fruit Products**	294.0 (241, 327)	68.5 (61, 289)	-	2.4 (2, 3)	0.4 (90, 1)	-	0.1 (0, 0)	0 (0, 0)	-	47.0 (38, 56)	17.5 (12, 57)	-	52.0 (12, 60)	10.0 (4, 120)	-
**Sugars and** **Sugar Products**	-	420.0 (301, 530)	529.0 (465, 550)	-	8.0 (0, 150	6.2 (4, 9)	-	0.1 (0, 3)	17.0 (7, 20)	-	47.0 (24, 59)	48.1 (38, 55)	-	24.0 (3, 40)	44.0 (12, 120)
**Beverages**	49.0 (45, 53)	-	44.0 (3, 50)	0.3 (0, 0)	-	0 (0, 0)	0 (0, 0)	-	0 (0, 0)	11.5 (11, 13)	-	7.9 (0, 12)	4.0 (0, 8)	-	8.0 (0, 20)
**Miscellaneous**	350.0 (348, 3710	77.0 (60, 102)	280.0 (115, 427)	44.6 (7, 51)	1.3 (1, 2)	3.3 (1, 6)	1.0 (1, 1)	0.1 (0, 0)	2.0 (0, 6)	10.0 (6, 11)	10.0 (5, 22)	3.3 (1, 11)	3995.0 (670, 7320)	720.0 (400, 980)	568.0 (352, 920)

Data presented as Median (Q1, Q3).

**Table 5:** Distribution in Nutri-Score grades of foods identified as NOVA4 based on the product name (generic) and products reclassified as NOVA4 after searching the ingredient list (originally grouped as NOVA1 or NOVA3 based on their name).

	Nutri-Score Grade *n* (%)				
	A	B	C	D	E
**NOVA4 from NOVA1**	73 (21.0)	139 (39.9)	46 (13.2)	25 (7.2)	64 (18.4)
**NOVA4 from NOVA3**	41 (8.9)	102 (22.0)	84 (18.1)	161 (34.8)	73 (15.8)
**NOVA4 generic**	228 (8.8)	665 (25.8)	448 (17.4)	634 (24.6)	602 (23.3)
**Total**	342 (10.1)	906 (26.7)	578 (17.0)	820 (24.2)	739 (21.8)

**Table 6:** Nutri-Score performance as FSAm-NPS score and Nutri-Score grades per food subcategory and per NOVA group as derived using both the product name and the ingredient list.

Food Category	Food Subcategory	NOVA Group	FSAm-NPS Score	*p*-Value	A [n (%)]	B [n (%)]	C [n (%)]	D [n (%)]	E [n (%)]
**Milk, Milk Product, or Milk** **Substitute (n = 1048)**	Milk (n = 174)	NOVA 1 (n = 94)	−0.117 ± 1.302	**<0.001**	42 (44.7)	52 (55.3)	-	-	-
		NOVA 3 (n = 6)	−1.667 ± 0.516		6 (100.0)	-	-	-	-
		NOVA 4 (n = 74)	1.595 ± 4.684		14 (18.9)	48 (64.9)	8 (10.8)	2 (2.7)	2 (2.7)
	Yogurt (n = 169)	NOVA 1 (n = 46)	−0.630 ± 2.886	0.853	22 (47.8)	15 (32.6)	9 (19.6)	-	-
		NOVA 4 (n = 123)	−0.447 ± 2.237		63 (51.2)	48 (39.0)	12 (9.8)	-	-
	Cheese (n = 213)	NOVA 3 (n = 127)	11.819 ± 7.140	0.125	3 (2.4)	26 (20.5)	5 (3.9)	84 (66.1)	9 (7.1)
		NOVA 4 (n = 86)	10.384 ± 7.288		6 (7.0)	17 (19.8)	12 (14.0)	46 (53.5)	5 (5.8)
	Milk Substitute (n = 410)	NOVA 1 (n = 6)	−1.000 ± 1.265	**<0.001**	4 (66.7)	2 (33.3)	-	-	-
		NOVA 3 (n = 54)	1.241 ± 3.923		5 (9.3)	46 (85.2)	1 (1.9)	-	2 (3.7)
		NOVA 4 (n = 350)	5.297 ± 8.564		63 (18.0)	167 (47.7)	30 (8.6)	33 (9.4)	57 (16.3)
	Milk Cream (n = 42)	NOVA 4 (n = 42)	8.191 ± 6.181	N/A	-	14 (33.3)	7 (16.7)	21 (50.0)	-
	Dairy Dessert (n = 40)	NOVA 4 (n = 40)	14.050 ± 6.341	N/A	1 (2.5)	3 (7.5)	2 (5.0)	23 (57.5)	11 (27.5)
**Fresh or** **processed eggs (n = 35)**	Fresh or processed eggs (n = 35)	NOVA 1 (n = 34)	−0.529 ± 1.107	**<0.001**	11 (32.4)	23 (67.6)	-	-	-
		NOVA 4 (n = 1)	−4.000		1 (100.0)	-	-	-	-
**Meat or meat product (n = 250)**	Meat analogue (n = 110)	NOVA 4 (n = 110)	5.818 ± 6.956	N/A	16 (14.5)	26 (23.6)	29 (26.4)	34 (30.9)	5 (4.5)
	Preserved meat (n = 83)	NOVA 1 (n = 1)	17.000	0.382	1 (100.0)	-	-	-	-
		NOVA 3 (n = 2)	17.000 ± 0.000		-	-	-	2 (100.0)	-
		NOVA 4 (n = 80)	10.463 ± 8.016		4 (5.0)	14 (17.5)	11 (13.8)	36 (45.0)	15 (18.8)
	Sausage or similar meat (n = 37)	NOVA 4 (n = 37)	11.027 ± 9.435	N/A	3 (8.1)	11 (29.7)	-	13 (35.1)	10 (27.0)
	Meat dish (n = 20)	NOVA 4 (n = 20)	7.300 ± 6.309	N/A	1 (5.0)	7 (35.0)	1 (5.0)	11 (55.0)	-
**Seafood or** **related product (n = 79)**	Seafood product (n = 79)	NOVA 3 (n = 54)	6.167 ± 6.624	0.463	3 (5.6)	19 (35.2)	11 (20.4)	20 (37.0)	1 (1.9)
		NOVA 4 (n = 25)	4.600 ± 7.427		6 (24.0)	9 (36.0)	4 (16.0)	4 (16.0)	2 (8.0)
**Fat or oil (n = 81)**	Vegetable fat or oil (n = 8)	NOVA 2 (n = 1)	0.000	**<0.001**	-	1 (100.0)	-	-	-
		NOVA 4 (n = 7)	15.429 ± 2.878		-	-	1 (14.3)	6 (85.7)	-
	Margarine or lipid of mixed origins (n = 39)	NOVA 4 (n = 39)	9.590 ± 3.618	N/A	-	1 (2.6)	21 (53.8)	17 (43.6)	-
	Butter or other animal fat (n = 34)	NOVA 2 (n = 32)	12.688 ± 8.042	0.343	-	8 (25.0)	1 (3.1)	7 (21.9)	16 (50.0)
		NOVA 4 (n = 2)	7.000 ± 9.899		-	1 (50.0)	-	1 (50.0)	-
**Grain or grain product (n = 1141)**	Cereal or cereal-like milling products and derivatives (n = 51)	NOVA 4 (n = 51)	7.059 ± 7.857	N/A	5 (9.8)	19 (37.3)	7 (13.7)	13 (25.5)	7 (13.7)
	Rice or other grain (n = 97)	NOVA 1 (n = 64)	−1.125 ± 1.741	**<0.001**	31 (48.4)	33 (51.6)	-	-	-
		NOVA 3 (n = 2)	4.000 ± 0.000		-	-	2 (100.0)	-	-
		NOVA 4 (n = 31)	6.516 ± 7.447		1 (3.2)	13 (41.9)	8 (25.8)	5 (16.1)	4 (12.9)
	Pasta and similar products (n = 202)	NOVA 1 (n = 167)	−2.934 ± 2.024	**<0.001**	161 (96.4)	4 (2.4)	2 (1.2)	-	-
		NOVA 3 (n = 9)	9.556 ± 5.897		1 (11.1)	1 (11.1)	2 (22.2)	5 (55.6)	-
		NOVA 4 (n = 26)	4.923 ± 7.579		10 (38.5)	2 (7.7)	7 (26.9)	7 (26.9)	-
	Breakfast cereals (n = 155)	NOVA 1 (n = 4)	1.250 ± 5.620	0.067	2 (50.0)	-	2 (50.0)	-	-
		NOVA 3 (n = 1)	10.000		-	-	1 (100.0)	-	-
		NOVA 4 (n = 150)	8.093 ± 5.836		16 (10.7)	15 (10.0)	59 (39.3)	60 (40.0)	-
	Bread and similar products (n = 259)	NOVA 4 (n = 259)	4.247 ± 6.865	N/A	43 (16.6)	114 (44.0)	53 (20.5)	35 (13.5)	14 (5.4)
	Fine bakery ware (n = 289)	NOVA 4 (n = 289)	14.948 ± 8.682	N/A	2 (0.7)	52 (18.0)	22 (7.6)	90 (31.1)	123 (42.6)
	Savory cereal dish (n = 88)	NOVA 4 (n = 88)	10.852 ± 5.247	N/A	1 (1.1)	7 (8.0)	22 (25.0)	53 (60.2)	5 (5.7)
**Nuts, seeds, or kernels (n = 130)**	Nuts (n = 68)	NOVA 1 (n = 20)	0.050 ± 6.485	0.215	14 (70.0)	2 (10.0)	2 (10.0)	1 (5.0)	1 (5.0)
		NOVA 3 (n = 16)	0.000 ± 3.983		6 (37.5)	4 (25.0)	6 (37.5)	-	-
		NOVA 4 (n = 32)	2.658 ± 6.136		9 (28.1)	9 (28.1)	11 (34.4)	2 (6.3)	1 (3.1)
	Seeds and kernels (n = 35)	NOVA 3 (n = 18)	12.000 ± 5.111	0.333	-	1 (5.6)	4 (22.2)	12 (66.7)	1 (5.6)
		NOVA 4 (n = 17)	10.471 ± 3.986		-	1 (5.9)	6 (35.3)	10 (58.8)	-
	Nut or seed product (n = 27)	NOVA 1 (n = 9)	11.000 ± 4.416	0.146	-	1 (11.1)	1 (11.1)	7 (77.8)	-
		NOVA 4 (n = 18)	13.833 ± 4.719		-	-	4 (22.2)	12 (66.7)	2 (11.1)
**Vegetable or vegetable product (n = 614)**	Vegetable (excluding potato) (n = 170)	NOVA 1 (n = 65)	−7.600 ± 2.416	**<0.001**	65 (100.0)	-	-	-	-
		NOVA 3 (n = 35)	−2.029 ± 7.127		27 (77.1)	3 (8.6)	1 (2.9)	3 (8.6)	1 (2.9)
		NOVA 4 (n = 70)	−3.729 ± 3.784		61 (87.1)	4 (5.7)	4 (5.7)	1 (1.4)	-
	Starchy root or potato (n = 21)	NOVA 1 (n = 1)	−2.000	0.984	1 (100.0)	-	-	-	-
		NOVA 3 (n = 2)	−1.500 ± 2.121		1 (50.0)	1 (50.0)	-	-	-
		NOVA 4 (n = 18)	−1.778 ± 2.463		10 (55.6)	8 (44.4)	-	-	-
	Pulse and pulse product (n = 423)	NOVA 1 (n = 423)	−6.742 ± 2.058	N/A	421 (99.5)	2 (0.5)	-	-	-
**Fruit or fruit product (n = 44)**	Processed food product (fruit) (n = 44)	NOVA 1 (n = 1)	1.00	0.941	-	1 (100.0)	-	-	-
		NOVA 3 (n = 5)	−0.200 ± 3.899		3 (60.0)	-	2 (40.0)	-	-
		NOVA 4 (n = 38)	0.316 ± 4.160		19 (50.0)	9 (23.7)	9 (23.7)	1 (2.6)	-
**Sugar or sugar product (n = 404)**	Sugar, honey, or syrup (n = 45)	NOVA 2 (n = 35)	0.000 ± 0.000	N/A	-	35 (100.0)	-	-	-
		NOVA 3 (n = 6)	0.000 ± 0.000		-	6 (100.0)	-	-	-
		NOVA 4 (n = 4)	0.000 ± 0.000		-	4 (100.0)	-	-	-
	Jam or marmalade (n = 83)	NOVA 4 (n = 83)	6.578 ± 5.808	N/A	2 (2.4)	27 (32.5)	21 (25.3)	32 (38.6)	1 (1.2)
	Non-chocolate confectionery or other sugar product (n = 68)	NOVA 3 (n = 22)	4.318 ± 7.767	0.020	8 (36.4)	6 (27.3)	-	7 (31.8)	1 (4.5)
		NOVA 4 (n = 46)	9.196 ± 7.921		9 (19.6)	6 (13.0)	2 (4.3)	28 (60.9)	1 (2.2)
	Chocolate or chocolate product (n = 208)	NOVA 4 (n = 208)	22.111 ± 6.589	N/A	-	7 (3.4)	10 (4.8)	19 (9.1)	172 (82.7)
**Beverage (n = 446)**	Juice or nectar (n = 163)	NOVA 1 (n = 48)	9.958 ± 2.073	0.138	-	-	2 (4.2)	17 (35.4)	29 (60.4)
		NOVA 3 (n = 1)	3.000		-	-	1 (100.0)	-	-
		NOVA 4 (n = 114)	9.588 ± 3.376		-	4 (3.5)	10 (8.8)	31 (27.2)	69 (60.5)
	Non-alcoholic beverage (n = 283)	NOVA 4 (n = 283)	8.371 ± 8.541	N/A	-	115 (40.6)	28 (9.9)	13 (4.6)	127 (44.9)
**Miscellaneous food product (n = 540)**	Spice, Condiment, or other Ingredient (n = 284)	NOVA 1 (n = 1)	15.000	N/A	-	-	-	1 (100.0)	-
		NOVA 2 (n = 1)	10.000		-	-	1 (100.0)	-	-
		NOVA 3 (n = 7)	2.714 ± 5.765		1 (14.3)	3 (42.9)	2 (28.6)	1 (14.3)	-
		NOVA 4 (n = 275)	9.851 ± 8.033		16 (5.8)	43 (15.6)	84 (30.5)	84 (30.5)	48 (17.5)
	Prepared food product (n = 172)	NOVA 4 (n = 172)	9.454 ± 6.758	N/A	8 (4.7)	28 (16.3)	45 (26.2)	74 (43.0)	17 (9.9)
	Ready-to-eat Food (n = 43)	NOVA 4 (n = 43)	1.349 ± 4.835	N/A	12 (27.9)	20 (46.5)	8 (18.6)	3 (7.0)	-
	Frozen, Semi-Ready Meal (n = 41)	NOVA 4 (n = 41)	−2.634 ± 6.335	N/A	28 (68.3)	7 (17.1)	4 (9.8)	2 (4.9)	-

## Data Availability

The original contributions presented in the study are included in the article, further inquiries can be directed to the corresponding author.
